# Supplementary material for: Evidence-Based Practices to Prevent Ventilator-Associated Pneumonia in an Intensive Care Unit in Bangladesh
Source: Healthcare (Basel). 2025 Nov 2;13(21):2782. doi: 10.3390/healthcare13212782 (PMC12610461; doi:10.3390/healthcare13212782)
Supplement: Supplementary file 1 [file healthcare-13-02782-s001.zip › healthcare-3910464-supplementary/Supplementary File Nahida.pdf]

Supplementary Table S1: Distribution of Micro-organisms in ventilated and clinically suspected VAP patients (Based on the number of specimens submitted)

Micro-organism	Tracheal C/S		Blood C/S		Total C/S	
	Control	Intervention	Control	Intervention	Control	Intervention
**All ventilated patients**						
Total	192	163	191	156	383	319
Positive	101	93	42	22	143	115
Acinetobacter	48	47	34	5	82	52
Candida	0	0	0	1	0	1
Citrobacter	0	1	0	2	0	3
Enterobacter	18	3	1	3	19	6
Escherichia	2	1	3	1	5	2
Klebsiella	11	18	1	3	12	21
Proteus	0	3	0	1	0	4
Pseudomonas	20	20	2	3	22	23
Staphylococcus	2	0	1	2	3	2
Others	0	0	0	1	0	1
Negative	91	70	149	134	240	204
Positive rate (%)	52.6%	57.1	22	14.1	37.3	36.1
**Clinically suspected VAP**						
Total	89	49	88	28	177	77
Positive	52	48	20	12	72	60
Acinetobacter	22	22	17	2	39	24
Candida	0	0	0	1	0	1
Citrobacter	0	1	0	1	0	2
Enterobacter	11	1	0	1	11	2
Escherichia	0	1	1	0	1	1
Klebsiella	5	10	0	2	5	12
Proteus	0	2	0	1	0	3
Pseudomonas	13	11	2	3	15	14
Staphylococcus	1	0	0	0	1	0
Others	0	0	0	1	0	1
Negative	37	1	68	16	105	17
Positive rate (%)	58.4	98	22.7	42.9	40.7	77.9

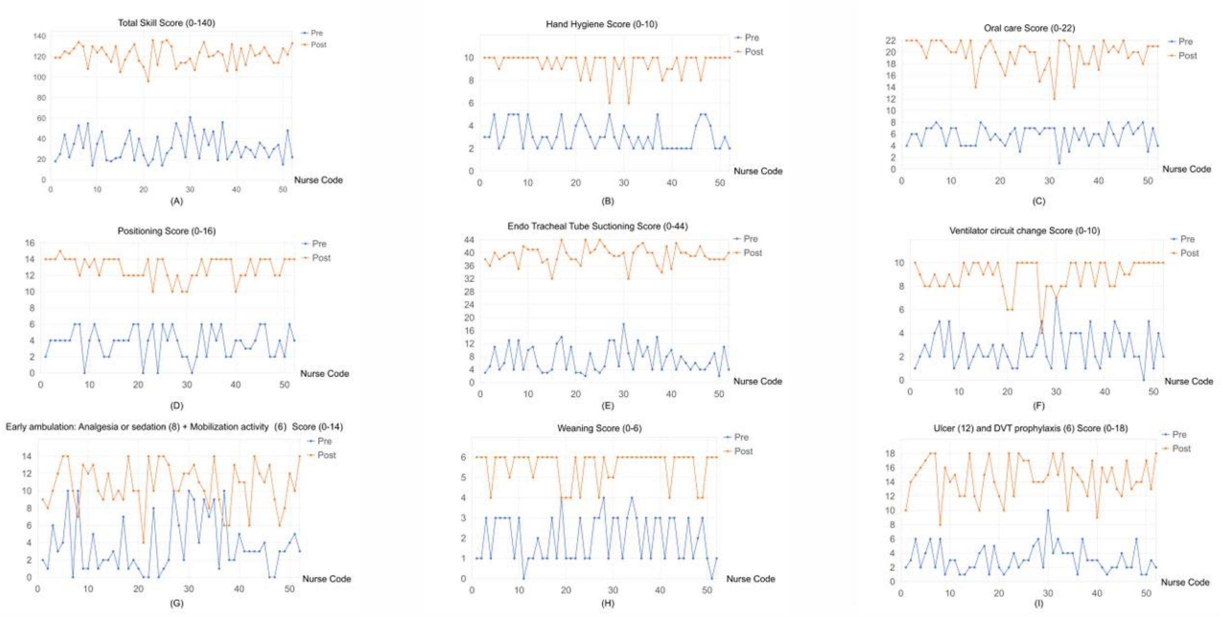

Supplementary Figure S1 nurses' VAP bundle skill improvement pre-and post-training
